# A Systematic Review: Migration of Chemical Compounds from Plastic Material Containers in Food and Pharmaceutical Fields

**DOI:** 10.3390/jox15060194

**Published:** 2025-11-11

**Authors:** Laura Culleré, Estela Sangüesa, Laura Lomba, María Pilar Ribate, Estefanía Zuriaga, Cristina B. García

**Affiliations:** Facultad de Ciencias de la Salud, Universidad San Jorge, Campus Universitario, Autov. A23 Km 299, Villanueva de Gállego, 50830 Zaragoza, Spainezuriaga@usj.es (E.Z.);

**Keywords:** migration, leaching, food, drugs, pharmaceutical, plastic containers, chemical compounds, phthalates, NIAS, endocrine disruptors

## Abstract

A systematic review was conducted on the migration of compounds from plastic containers in the food and pharmaceutical industries, using Web of Science databases and following PRISMA guidelines (Preferred Reporting Items for Systematic Reviews and Meta-Analyses). The protocol has been registered with the OSF registry, with the DOI 10.17605/OSF.10/UQ3T2. This review included only review articles in English published within the last fifteen years. Four reviewers independently screened titles and abstracts, discussing inclusion criteria. In this comprehensive evaluation of the information present in an Excel spreadsheet, a substantial number of records were discarded because they were not representative of the topic under study. Following the review process, a total of twenty-eight key records were selected, primarily focusing on migration in the food and pharmaceutical sectors. Of these, twenty-four addressed only food, just two addressed only pharmaceutical sector, and two covered both fields, highlighting limited information on migration in pharmaceuticals, cosmetics, and related products. The analysis emphasized the types of compounds studied, the analytical methods employed, the migration tests conducted, and the toxicity assessments undertaken. The most frequently assessed compounds included phthalates, endocrine disruptors like bisphenol A, and non-intentionally added substances (NIAS). Analytical methods used typically involved pre-treatment steps, such as liquid–liquid or solid-phase extraction, followed by gas or liquid chromatography, depending on compound volatility.

## 1. Introduction

### 1.1. Plastic Containers, Advantages and Disadvantages for Their Uses

Plastic is a material that is frequently used for the purpose of packaging pharmaceuticals and food products. This is due to its excellent properties such as flexibility and light weight. The major components of plastics (70–99%) are polymers. Some of the most used polymers are based on polyethylene (PE), polyamide (PA), polypropylene (PP), and polyvinylchlroride (PVC). Moreover, plastics contain macromolecules and additives such as antioxidants, stabilizers, dyes, and lubricants, which improve the properties of plastics, increasing their flexibility, elasticity, softness, and reducing their fragility [1].

Plastic materials offer several advantages when used for packaging. Firstly, the lightweight nature of plastic packaging reduces fuel consumption and lower greenhouse gas emissions during transportation compared to heavier materials such as glass or metal. Additionally, plastic is relatively inexpensive to produce, making it a cost-effective choice for manufacturers and consumers alike. It is highly durable and impact resistant, protecting against moisture, light, and gases; it offers a high resistance to water and air, a characteristic that enhances its suitability for safely storing sensitive products, including pharmaceuticals and food items [2]. Moreover, the versatility of plastic further allows for the design of packaging in a wide range of shapes and sizes.

However, the use of plastics not only entails advantages but also presents negative aspects that must be considered. One of the most relevant problems is their negative impact on the environment and ecological systems [3]. Alternatives of common plastics are recycled ones. In the EU, this kind of plastics reached more than 10 million tons in 2021, but chemical deterioration during recycling has been observed [4]. Moreover, bioplastics manufacturing is growing at a rate of 10% per year. This term “bioplastics” is used to refer to plastics that are biodegradables or can be bio-based, or both [5]. The most used bioplastics are polylactic acid (PLA), with similar characteristic to polystyrene; polyhydroxyalkanoates (PHAs), with similar properties to PE and PP; and polybutylene succinate (PBS), very similar to PP. The impact of these alternative materials on the environment is a matter of great concern.

One of them is the fact that many additives, which are required to enhance the properties of plastics, are highly toxic and can therefore be harmful to health at certain exposure levels. This is the case for families such as phthalates, brominated flame retardants, and chlorinated paraffins. These compounds are all regarded as toxic, carcinogenic, and mutagenic, and some are also endocrine-disrupting substances [6]. Another important aspect is that many of these additives, such as phthalates—also known as phthalic acid esters or phthalate esters—can easily migrate from the material since they are not chemically bound to the polymer matrix. In addition, plastics may also contain residual solvents and catalysts from their synthesis, as well as impurities, degradation products, and non-intentionally added substances (NIAS) [7].

### 1.2. Additives Used in Plastic Materials, Risk Assessment of Health Human

Phthalates are a family of compounds that are usually added to PVC to make it hard. Moreover, phthalates are used in the polymerisation processes of polypropylene (PPE), polyethylene (PE), and polystyrene (PS) as part of the catalyst [8]. Therefore, phthalates can be found in both PVC and non-PVC materials that come into contact with food and pharmaceuticals [9,10]. The most commonly used phthalates are dimethyl phthalate (DMP), di-n-butyl phthalate (DnBP), benzyl butyl phthalate (BBP), diethyl hexyl phthalate (DEHP), di-n-octyl phthalate (DOP), diisononyl phthalate (DINP), and diisodecyl phthalate (DIDP). Among all of them, DEHP is considered as the principal plasticizer used in medical devices and pharmaceutical contact materials [11]. However, DEHP is classified as carcinogenic, mutagenic, or toxic to reproduction (CMR 1B) under the regulation on the classification, labelling, and packaging of substances and mixtures, (CLP Regulations) [12], and as a consequence, the use of this phthalate was restricted and there has been interest in non-DEHP alternatives, for example, in the case of PVC medical devices.

Many studies have shown that exposure to phthalates may cause reproductive toxicity, liver damage, and carcinogenesis in humans [13,14,15,16,17,18,19]. DnBP, DEHP, BBP, DIDP, and DINP are the main dangerous phthalates in terms of human health, considered as endocrine disruptors. Therefore, it is important to monitor their presence in food and pharmaceutical industries. For this reason, European Food Safety Authority (EFSA) established these tolerable daily intakes for these phthalates: 0.01, 0.05, and 0.5 mg/kg body weight per day for DnBP, DEHP, and BBP, respectively, and 0.15 mg/kg body weight per day for DINP and DIDP [20]. As already shown, because of their low interactions and no chemical bonding with polymer chains in polymer matrices, phthalates are likely to migrate from plastics into a medium with which they come into contact.

In the case of polyethylene (PET) plastic materials, it is well known that a family of oligomers are ubiquitous Therefore, migration of these oligomers from PET used as food contact materials has been widely reported in the literature [21].

Regarding bio-based and biodegradable materials, according to a study published by Zimmermann et al. [22], they showed a similar chemical compounds profile, including additives, lubricants, intermediates, etc., to that found in polyvinyl chloride and polyurethanes. These results suggested that these alternative materials are not necessarily safer than conventional plastics.

In general, the potential toxicity of migrant compounds to humans and animals is a matter of great concern, mainly for the food and drug industries [23,24].

There are several potential exposure pathways of phthalates in humans, including ingestion, inhalation, skin absorption, and intravenous injection. Human exposure to these chemical compounds can occur after contact or use of a product containing them, or through the leaching of these substances from packaging in general [25].

### 1.3. Leaching or Migration Processes, Consequences in Food and Pharmaceutical Fields Area

Although the concern is common, in the food safety field the term “migrant” is commonly used to label these compounds, while in pharmaceutical area, the term “leachable” is most used. Leachables were initially defined as compounds that leach into the formulation from elastomeric or plastic compounds of the drug product container closure system [26]. However, a more generic definition is any chemical species that migrates from a drug contact material into the drug products during manufacturing, storage, distribution, or clinical use of the product under normal conditions.

The leaching or migration process is typically undesirable, as it has the capacity to compromise the properties of the plastic material. This, in turn, can result in the contamination of consumer products, particularly in the context of food contact materials, pharmaceuticals, and medical devices [27,28]. Migration of chemical substances in food or medicine plastic packaging are examples of undesirable migration, due to the fact that these compounds may be toxic [29,30], give an unpleasant taste to the food, or deteriorate the medicine, degrading the active substances and modifying their therapeutic action [27].

In the case of plastics in contact with food (food contact materials, FCMs), the migration process is favoured when the packaging is subjected to heat sources, such as microwaves or ovens [31]. In the domain of pharmaceuticals, the impact of heat on migration has not been a subject of evaluation, primarily due to the storage conditions of these products, which typically occur at room temperature or under refrigeration.

Therefore, in both areas (food and pharmaceutical industry) it is very important to control the range of additives and the migration/leaching processes to ensure the safety to the public health [6,32].

However, in comparison with the foodstuffs area, there are very few regulations covering drug leachables, and only the US Pharmacopeia has a specific monograph on this subject [33].

The aim of this review was to systematically explore the studies published about the migration process from plastic material containers in food and pharmaceutical industries and compare both results. This review systematically investigates the migration or leaching processes of chemical compounds from plastic containers in food and pharmaceutical sectors, focusing on analytical methods, toxicological risks, and regulatory frameworks. It could find research gaps to propose advancements to homogenize safety standards.

## 2. Methods

The review process was conducted in line with the Preferred Reporting Items for PRISMA 2020 checklist (Appendix A) [34]. This protocol has been submitted and registered with the Open Science Framework (OSF, https://osf.io/, accessed on 5 November 2025). The registration DOI is 10.17605/OSF.10/UQ3T2, accessed on 5 November 2025.

### 2.1. Search Strategy

The search queries were conducted independently in the electronic database Web of Science using the same keywords, but with one difference based on the final fate of plastic components (food or pharmaceutical field). The review protocol was specifically prepared to be accessed on OSF registries with the DOI number 10.17605/OSF.10/UQ3T2, accessed on 5 November 2025.

The general keywords on TOPIC search were as follows: (migra* OR leach*) AND (plastic*) AND (determination OR quanti*). In addition, the specific keywords were lastly added: AND (food OR drink*) in the case of food search or AND (drug* OR medic* OR cosmetic* OR pharmaceutic*) in the case of pharmaceutical field search up to 27 August 2025.

### 2.2. Inclusion and Exclusion Criteria

Articles were included in the systematic review if (1) they were published within the last fifteen years (from 2010 included), (2) categorized as review articles, and (3) published in English language. The decision to include solely review articles was primarily influenced by the substantial volume of entries that would otherwise be encountered if the original articles were also included. Moreover, the main advantage of this type of articles is that they compile the previous evidence of what has been published on the subject and provide a more global view of the situation of the topic.

### 2.3. Study Selection

Titles and abstracts of the selected articles were screened by four reviewers (C.B.G., M.P.R., E.S., E.Z.) independently. Those articles that did not correspond to the topic of the study were discarded directly by these reviewers. A joint meeting of all reviewers together with L.C. and L.L. was held to discuss the articles that raised doubts and to share the causes of discarding some papers. Duplicate articles from the two searches were considered for both studies if they were informative for both topics, as decided in the meeting. The full manuscript text of these potentially eligible articles was retrieved and assessed by the same investigators for inclusion in the review.

### 2.4. Data Extraction

A standardized table in Microsoft Excel was used for data extraction from the included articles by the reviewers (C.B.G., M.P.R., E.S., E.Z.). The table covered the following information: study name, authors, year of publication, DOI number, analytes or families of compounds studied (including concentration data), methods of analysis (if available, with detection limits), variables taken into consideration such as temperature or humidity, and study findings.

### 2.5. Data Quality Assessment

The utilization of a colour-coding system was used to assess the quality of each study from red (high risk of bias) to green (low risk of bias) in the Excel table. In case of concern, the study was awarded the colour yellow, and these findings were resolved by group consensus in a meeting (four reviewers and L.C. and L.L.). Bias was considered if the study did not show clearly the origin of data, analyzing method, or had confusing or non-well-defined results or conclusions.

### 2.6. Data Analysis/Synthesis

Collected data were shared and discussed in a consensus group including the four reviewers and L.C. and L.L. Only results from non-biased papers (green and yellow) were considered for final results of the study.

## 3. Results and Discussion

### 3.1. Search Results and Included Studies

**Figure 1:**
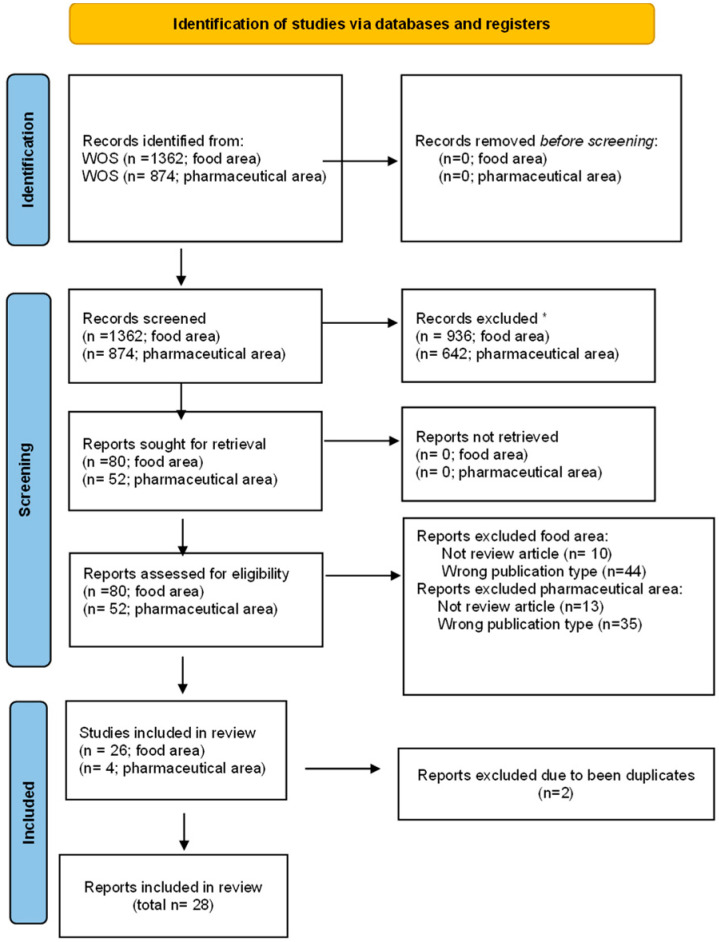
Prisma flow chart [34]. Description: Pictorial diagram of the process used for identifying and screening relevant records for inclusion, following PRISMA guidance. * Records excluded because they were not published during the last fifteen years, they are not reviews, or they are not written in English language.

As a result of the initial search in the food sector, 1362 records were found, of which 936 had been published within the last 15 years (meeting the initial inclusion criterion). Therefore, the present study comprised works that were published from the year 2010 onwards. The next criterion that was applied was to include solely those studies that were published in the form of a review. This process resulted in a total of 82 works, of which according to WOS, 80 were written in English (this was the subsequent inclusion criterion), as it is shown in Figure 1.

Similarly, the search was conducted for the pharmaceutical field. In this case, 874 records were found, which is a lower number when compared to the 1362 found for food, of which 642 had been published in the last 15 years (first inclusion criteria). The next criterion was to include only studies published in the form of reviews, and 54 records were found, again significantly fewer than the 82 found in the food sector. Of the 54 records under consideration, 52 were written in English, as Figure 1 shows.

Had a final criterion of inclusion been applied to include reviews published exclusively in open access, there would have been only 32 and 24 records in the fields of food and pharmacy, respectively. This would have resulted in a significant loss of information.

In conclusion, 80 records related to studies on the migration or leaching process from plastic materials in the food industry and 52 records from the pharmaceutical industry were selected for inclusion in the final analysis (Figure 1).

With these included studies, a database was created using Excel, where the relevance of the title and abstract was assessed. Additionally, in order to ascertain the efficacy of the other inclusion criteria applied to the searches, a verification process was undertaken.

In this comprehensive evaluation of the information present in the Excel sheet, a considerable number of records were discarded on the basis that they were not representative of the topic under study.

In the context of migration within the domain of food, out of the 80 records that were incorporated into the study, 10 were discarded because they were not reviews (despite having applied the corresponding filter in the WOS search), and the remaining 44 were excluded because the title reflected a research topic that did not align with the focus of this work. Most of these reviews addressed contamination caused by the presence of microplastics and nanoplastics in the environment (mainly soils); others focused on studying families of compounds (such as phthalates, BPs) or individual compounds (like BHT) but were not related to migration topics.

In the case of the pharmaceutical field, the exclusion of previously selected reviews was due to analogous reasons. Thirteen of them were not reviews, and among the remainder, many dealt with issues related to environmental pollution and ecotoxicity resulting from microplastic presence. Furthermore, a significant percentage focused on topics related to health and diseases such as migraines, kidney injuries, central nervous system disorders, depression, etc.

Therefore, after evaluating the relevance of these 80 and 52 records, the number was significantly reduced to just 26 and 4, respectively. All are clearly reviews, except for one, which is a commission report on the health impact of plastic migration in both sectors [35].

A total of 28 records have been finally included in this study. Of these, 24 correspond to publications exclusively in the food field, 2 to the drugs field, and the remaining 2 refer to both the food and pharmaceutical sectors [35,36].

### 3.2. Study Characteristic

A summary of the characteristics of included studies can be found in Table 1.

The main compounds or family of compounds analyzed in the included studies are phthalates, NIAS, and endocrine disrupting chemicals such as bisphenol A.

Regarding the topics discussed, sixteen of them comment on risk assessment, twenty-four provide insights on the different analytical techniques applied for the quantification of the compounds of interest, and nine of them comment on migration testing.

#### 3.2.1. Toxicity Risk Assessment

Cramer’s rules are a classification system that assigns substances to different risk categories, primarily by implementing the TTC (threshold of toxicological concern) concept. Nevertheless, the assessment of genotoxicity remains more complex and represents a significant challenge [55]. The Cramer decision tree, subsequently refined by EFSA, enables the classification of substances into three toxicity classes (I, II, or III) based on their chemical structure, the presence of functional groups, and the potential for metabolic activation. These factors may serve as indicators of toxicological concern.

- Class I (low toxicity): Encompasses compounds with simple chemical structures and efficient metabolic pathways, which are indicative of a low toxic potential. A TTC threshold value of 30 µg/kg body weight/day is assigned. Typical examples include sugars and amino acids.
- Class II (intermediate toxicity): Includes compounds with moderately complex chemical structures. The TTC threshold is set at 9 µg/kg body weight/day. Alcohols and esters are representative of this category.
- Class III (high toxicity): Comprises compounds whose structural features suggest potential toxicity, including aromatic substances. These are assigned the most conservative TTC threshold value of 1.5 µg/kg body weight/day.

Despite the extensive implementation of the TTC-based approach, it is important to acknowledge its limitations, particularly for bioaccumulative or genotoxic substances. In such cases, Commission Regulation (EU) No 10/2011 establishes a more stringent specific migration limit of 0.00015 mg/kg.

Schreirer et al. [21] studied polyethylene terephthalate oligomers, considered as non-intentionally added substances (NIAS) and ubiquitous in PET food contact materials (FCMs). One approach to the assessment of oligomers is commonly carried out through the evaluation of their corresponding monomers, under the assumption of 100% hydrolysis of the former. Accordingly, in the case of PET, the levels of its monomers are assessed, namely ethylene glycol (EG), terephthalic acid (TPA), and isophthalic acid (IPA). These monomers are subject to specific migration limits of 30 mg/kg, 7.5 mg/kg, and 5 mg/kg, respectively [61]. An additional difficulty with respect to oligomers is that their identity, level of migration, and toxicological characteristics are often unknown [62]. The use of the threshold of toxicological concern (TTC) concept is a strategy used to estimate safety values for PET oligomers of unknown toxicity. However, it is not clear whether the use of this concept is appropriate for PET oligomers [63].

Despite all the data provided, there is an important gap in toxicological research, and no hazard assessment can be performed.

The European Food Safety Authority (EFSA) has distinguished substances depending on their level of migration. In the case of high compound migration levels (ranging from 5 to 60 mg/kg of food), a comprehensive array of toxicological data is required. This should include an in vitro genotoxicity test; a 90-day oral toxicity study; studies on absorption, distribution, metabolism, and excretion; studies on reproduction; and studies on long-term toxicity and carcinogenicity.

When the substance is migrating within the range of 0.05–5 mg/kg of food or food simulant, a minimum of two genotoxicity tests are required. Furthermore, a 90-day oral toxicity study and data to evaluate the absence or presence of potential for accumulation in humans. Finally, in the case of low levels of migration process, corresponding to levels lower than 0.05 mg/kg of food or food simulant, the toxicological data required is considerably reduced (only two genotoxicity tests are needed).

However, only intentionally added substances are subjected to EFSA authorization in the European Union. Therefore, non-intentionally added substances (NIAS), such as impurities, and degradation of products during the production of plastic materials, solvents, among others, are not included in the EU list.

As illustrated in the relevant literature, certain **NIAS** have been identified in food contact materials. These include primary aromatic amines from polyureth adhesives, as well as breakdown products such as carbonyl compounds like nonanal, glyoxal, methylglyoxal from PET, and different contaminants from recycling processes, including PET oligomers or diethylene glycol dibenzoate from recycled PET [64,65,66]. In such cases, companies must be able to assure the absence of risk to human health, based on internationally recognized scientific protocols or principles. Nevertheless, the implementation of these principles is challenging.

In the case of NIAS risk assessment, the most widely employed instrument is the threshold of toxicological concern (TTC) [67,68,69]. The use of the TTC concept was proposed for regulating the presence of NIAS. However, the identification of these substances, particularly those with genotoxic potential, remains an unresolved issue. The migration level at which a safety assessment of NIAS compounds becomes necessary can be established on the basis of either the detection limit of the analytical technique employed or the safe exposure threshold [70].

**Bisphenol A (BPA)** and its derivatives are the main components of polycarbonate (PC) plastics and epoxy resins. Consequently, they have been widely used in the production of packaging materials, and it is probable that they will be detected in food contact materials (FCMs). In 1993, Krishnan and coworkers [71] demonstrated for the first time that BPA migrated from PC plastic under certain temperature conditions and that it also exhibited endocrine activity. According to the European Food Safety Authority (EFSA) and Commission Regulation (EU) 2018/213 of 12 February 2018, the tolerable daily intake of BPA is 4 µg/kg body weight per day, and the specific migration limit (SML) of BPA is 0.5 mg/kg [44].

Regarding **phthalic acid esters**, considered as toxic and a potential risk to human health, it is possible to distinguish between them depending on their polarity and long-chain. Due to the health risks associated with certain authorized phthalates, namely DEHP, BBP, DBP, DINP, and DIDP, the European Food Safety Authority (EFSA) established several years ago a tolerable daily intake (TDI) of 50 µg/kg body weight per day for all except DIDP, for which a TDI of 150 µg/kg was set [17].

**Other class of additives** that are capable of protecting plastics from degradation by oxidation, ozone, heat, light (including UV), and bacterial attack are UV stabilizers [6]. Benzotriazoles are UV stabilizers that are present in plastic bottle caps and food packaging [72]. The Food Packaging Forum Foundation [70] has demonstrated that a minimum of nine benzotriazoles migrate from plastic food contact material. These compounds exhibit a high degree of lipophilicity and bioaccumulation and have been detected in human blood, urine, and breast milk [72].

Nonylphenols, with endocrine-disrupting properties, are used as antioxidants in various resins [70]. It has been demonstrated that these compounds together with bisphenol A also migrate from bottles into the water they contain, a phenomenon that has been observed for PVC bottles and caps [73,74,75,76].

**In the pharmaceutical industry**, the levels of potentially toxic leachables are also monitored under accelerated stability conditions [28]. The pharmaceutical guidelines establish a Safety Concern Threshold (SCT) depending on the route of administration: for oral, nasal, or inhalation products, the SCT is set at 0.15 µg/day, while for parenteral or ophthalmic products, the SCT is elevated to 1.5 µg/day [31].

In medical devices, this phenomenon of migration must also be considered, given its potential impact on human health [9,57,77]. In the case of DEHP, this compound cannot be used in the manufacture of such devices due to its high toxicity, and alternative plasticizers have had to be sought [78].

#### 3.2.2. Analytical Techniques

**Figure 2:**
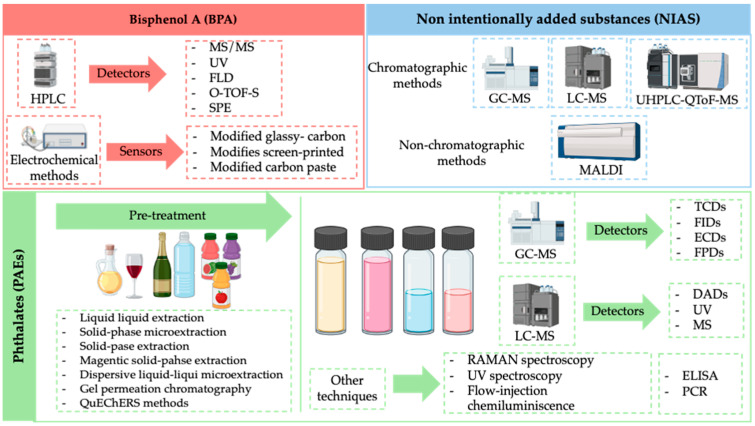
Analytical techniques used for determination of bisphenol A, non-intentionally added substances and phthalates. **Description:** This figure schemes all the information related to the different possibilities of analytical strategies to determine the different analytes or family of analytes of interest (bisphenol A, NIAS and phthalates). This figure includes both pre-treatment strategies and analytical techniques.

The analytical techniques and pre-treatment steps used to determine the different substances are shown in Figure 2.

All analytical methods compiled by Priovolos et al. [44] to determine bisphenols are based on **HPLC**. They are simple, sensitive, fast, and easy to be used. The differences between the different methodologies proposed consisted in the type of detector coupled with HPLC technique MS/MS [74,75] UV [76,77,78], fluorescence detector (FLD) [79], Q-TOF-MS [80], the sample pretreatment strategy (SPE) [74,81,82], and dispersive solid phase extraction (DSPE) [80]. In general, limit detections reached were very low values. For example, Lian et al. [80] obtained a limit of detection of only 0.08 µg/L of BPA using DSPE-HPLC-QTOF-MS in plastic food packaging materials samples, and Choi and coworkers [77] obtained a LD of 3.05 µg/L using magnetic DSPE-HPCL-UV in milk samples.

Gugoasa et al. [41] focused on the electrochemical methods and sensors used for the quantitative determination of bisphenol A. In the recent years, electrochemical sensors used for the determination of BPA [83,84,85] have been shown to be an alternative to classical methodologies due to their good selectivity, high sensitivity, and no required pre-treatment step. The field of electrochemical sensors is subdivided into three distinct categories: modified glassy-carbon electrodes, modified screen-printed electrodes, and modified carbon paste electrodes. The choice of the type of sensors depends on the applications for which it is to be employed.

Depending on the volatility of the predicted NIAS compounds, **GC-MS** is applied for highly volatile substances, whereas GC-MS and LC-MS are employed for semi-volatile and non-volatile compounds [47]. In a recent publication by Aznar et al., a strategy was presented for the quantification of non-volatile migrants from a polyester material based on UHPLC-QToF-MS [26]. 

Attending to **non-chromatographic techniques** for NIAS determination, ambient mass spectrometry (AMS) methods [86,87] and matrix-assisted laser ablation (MALDI) [88] have been proposed for migrating solutions.

In gas chromatography, alternative detector mechanisms encompass thermal conductivity (TCDs), flame induction detectors (FIDs), electron capture detectors (ECDs), and flame photometric detectors (FPDs). GC-FID has limited selectivity and specificity compared with GC-MS methods [89,90]. Chromatographic techniques including gas chromatography (GC) and liquid chromatography (LC) followed by mass spectrometry detection are frequently used to quantify phathalates and to obtain the most comprehensive profiling of NIAS released [91,92,93,94].

Conversely, the detectors commonly used for the detection of PAEs in edible oils using LC included DADs, ultraviolet (UV) detectors, and MS detectors (MS, MS/MS). In comparison with LC-UV or DAD, LC-MS/MS is more selective and reliable for the quantification of phthalates in a shorter analysis time [43].

According to the review published by Freitas et al. [50] in which compiled studies that quantified this family of compounds in olive oil and wine matrices, more than 90% of these papers used chromatographic strategies (25% applied LC and 67% applied GC).

However, it should be noted that there are also **other analytical approaches to chromatography**, which can likewise be applied in these types of analyses. For instance, the following methodologies may be employed: Raman spectroscopy, flow-injection chemiluminescence (FI-CL), ultraviolet spectrophotometry (UV), or strategies based on enzyme-linked immunosorbent assay (ELISA) and polymerase chain reaction (PCR).

In a literature review carried out in 2019 by Sica et al. [31], mass spectrometry was highlighted as a powerful and widely used detector in the analysis of extractable and leachable compounds within the pharmaceutical industry. It is worth noting that each modification of a pharmaceutical formulation can result in alterations to the leachable profile and the compounds that may migrate or interact with the packaging. Consequently, research in this field has intensified due to the potential health implications associated with the presence of certain compounds. For this reason, it is expected that, in the coming years, new methodologies and protocols will be developed to assess the risk posed by these migrating compounds 91.

Chromatographic methods coupled with mass spectrometric detection are considered the reference and most widely applied techniques in the study of migration, as well as in the characterization of impurities and active pharmaceutical ingredient (API) degradation products. One of the main reasons is their high sensitivity, which enables the quantification of trace concentration levels (µg/L), in addition to their high selectivity. However, in some cases, such as in the analysis of phthalates, a preconcentration step is required prior to analysis [74,80,81,82]. Several pretreatment approaches have been reported in the literature, including liquid–liquid extraction (LLE), a technique frequently used for the analysis of phthalates in olive oils and wines [50], solid-phase microextraction (SPME) [50], and solid-phase extraction (SPE) [81,82].

It is acknowledged that there are a number of less frequently employed strategies that may nevertheless be of interest in this context. These include the use of molecularly imprinted polymers in solid-phase extraction (MISPE) and magnetic solid-phase extraction (MSPE), as well as dispersive liquid–liquid microextraction (DLLME). Gel permeation chromatography (GPC) and *quick, easy, cheap, effective, rugged, and safe* (QuEChERS) methods have also been applied, among others [50].

LLE strategy is still used in many cases, although this has several limitations. These include the presence of potentially harmful solvents in significant volumes and the fact that it is a time-consuming process. For the extraction from edible oils, the commonly used extraction solvents are mainly nonpolar solvents such as n-hexane, isooctane, acetonitrile, and dichloromethane. While dichloromethane is effective for extracting non-polar compounds, its high volatility and toxicity limit its routine use. Conversely, acetonitrile offers broader polarity coverage but may be less efficient for extracting hydrophobic NIAS [59]. LLE followed by GC-MS/MS detection reached limits of detection in the range of 0.1–4 µg/kg of edible oil samples for phthalates analyzed [91]. On the other hand, SPE and SPME strategies have demonstrated better characteristics, reaching higher extraction efficiency and also higher enrichment levels of analytes. Alternative strategies such as QuEChERS, proposed for the first time by Anastassiades et al. in 2003 [92] also have been used. Some of the main advantages for this strategy are its simplicity, low solvent consumption and flexibility. QuEChERS has recently emerged as a novel approach for the detection of trace organic compounds in foods, with a wide range of application in FCMs [93,94].

#### 3.2.3. Migration Tests

The phenomenon of migration can be influenced by several factors, including the nature of the polymeric material, the type of product contained, the temperature and duration of contact, as well as the properties of the migrating chemical compounds and the type of polymer. Each plastic material has different permeability and affinity toward the potential migrant compounds.

For migration and extraction testing, simulant methods and migration models are typically employed, with the application of severe conditions [47,61]. A limitation of this approach is that it does not permit the prediction of NIAS migration.

According to EU Regulation No. 10/2011, the food sector is subject to two distinct categories of migration tests. Firstly, it is understood to mean ‘the maximum permitted amount of non-volatile substances released from a material into simulants under specified conditions’. Secondly, specific migration is understood to mean ‘the maximum permitted level of a given substance that can migrate into the actual product or into food simulants’ [61]. The significance of monitoring potential migrating substances in the food sector is such that, in the United States, the FDA requires companies to estimate the daily dietary intake of migrating substances using food simulant models.

The migration of PET oligomers from FCMs has been widely studied, and Commission Regulation (EU) 10/2011 establishes a framework for conducting migration tests. However, Schreirer et al. [21] concluded that the reported data on this topic was heterogeneous, using different conditions and different methodologies, making it difficult to compare it and draw general conclusions. Using ethanol–water mixtures as a food simulant, as regulations indicates, is not appropriate for determining the migration of PET oligomers.

In the case of bisphenol A migration from FCMs, numerous studies have focused on it. The most significant parameters that can affect this migration are the temperature, the pH value and the contact time as well. Moreover, the presence of alkaline detergents, which can remain on the surface of plastic bottles that increase the migration of BPA, and the presence of impurities and amines, can also accelerate the hydrolysis of BPA at high temperatures [44]. It is clearly observed that the BPA concentration is increased by the temperature.

In a review by Urbelis et al. (2021) [53], it is noted that depending on the type of packaging material used—whether paper, cardboard, or plastics—the choice of food simulants varies, and testing is always performed under the worst-case conditions. This phenomenon can be attributed to the fact that the migration of a substance is predominantly influenced by the physicochemical properties of the migrating compound and those of the food. Some relevant properties include volatility, molecular structure, and food composition. Environmental factors such as temperature and humidity also play an important role. According to Commission Regulation (EU) No 10/2011, for migration studies from plastics into dry foods, Tenax must be used as the food simulant [55,61].

For aqueous and acidic foods, an aqueous simulant is recommended, which may contain 10% ethanol, 3% acetic acid, or alternatively, 20% ethanol. Testing is typically conducted over 10-day storage periods at temperatures ranging between 40 and 60 °C. 

Furthermore, different migration testing studies have been published. The conditions of each study depend on the legislation and regulations of each state. Some of the results obtained are obtained by Wang et al. [95]. These authors conducted a study on the migration of BPA from plastic packaging to meat products and demonstrated that the ratio of migration increased in a linear way with storage time. In accordance with the findings of the study, plastic packaging is considered as the main source of BPA contamination in non-canned meat products [95].

Zhou et al. [76] also demonstrated that levels of BPA migration increased to both storage time and temperature. Park et al. [96] evaluated the level of BPA migration from polycarbonate baby bottles using four different types of food simulants. The study revealed that BPA migrated in a higher ratio into n-heptane at 25 °C and in the second place using a 50% ethanol–water solution at 70 °C. However, using water and a 4% acetic acid solution was not effective to migrate BPA at a detectable level [96].

The NIAS assessment in plastics FCMs is usually made by migration test under worst-case conditions using food simulants [52]. It is noteworthy that while EFSA sets maximum migration limits (SML values) and tolerable daily intake (TDI values) for migrating compounds in general, there are no such regulations for the family of NIAS. This creates a gap in the legislation resulting from a lack of information related to the nature of this group of substances.

It is also interesting that studies carried out on biopolymers (PLA-based films) revealed cyclic lactide oligomers and adipate derivatives as the predominant migrant, using migration tests simulating both fatty food and aqueous conditions [97].

In conclusion, it has been taken into account that among all the factors, temperature is considered as the key factor that determines the migration of chemical compounds.

## 4. Discussion

### 4.1. Key Findings

When analyzing the review articles published over the years, it can be seen that 80 reviews have been obtained for food area and 52 reviews for drugs. In both cases, there has been an increase over the years. The reviews that have been published since 2020 to the present day account for approximately 70% of the total, indicating a growing interest in the issue of migration in both areas under study.

Regarding the most commonly used analytical strategies for determining these migrating compounds, although new non-chromatographic analysis methods are increasingly being researched and proposed, there is no doubt that chromatographic techniques remain the most widely used (LC for low volatile and heavy compounds and GC for volatile and light ones). These techniques offer a wide range of detectors, with the most selective ones, such as those based on mass spectrometry (MS, MS/MS, etc.), being preferred.

As for sample preparation strategies, they are essential for achieving both adequate selectivity and sensitivity. Many alternatives to traditional methods, such as liquid–liquid extraction (LLE), are being proposed, but LLE is still in use. It is true that solid-phase extraction (SPE), solid-phase microextraction (SPME), and other alternatives that require less organic solvent and reagent use, in addition to diminished levels of sample manipulation, thereby facilitating enhanced automation, are progressively preferred.

The analytes or families of analytes identified as migrating compounds in this work are primarily bisphenol A, phthalates, and NIAS. It is noteworthy that while EFSA establishes maximum migration limits (SML values) and tolerable daily intake (TDI values) for migrating compounds in general, there are no such regulations for the family of NIAS. This creates a gap in the legislation, resulting from a lack of information related to the nature of these substances.

It is imperative to acknowledge that these types of substances, which are generally found in packaging, toys, clothing, and other items, can be absorbed and introduced into the body through different entry routes. This underscores the necessity for further comprehensive studies to be conducted, with the aim of raising awareness about the potential hazards posed by these substances in everyday items.

### 4.2. Future Policy and Research

Based on the conducted search, focusing only on the reviews, there is a clear disparity in publications related to the migration of chemical compounds in the food field area compared to the pharmaceutical area. Only two records finally selected for this study uniquely address this migration process in the pharmaceutical field.

The issue is the same in both areas, as the compounds migrating or leaching from plastics can significantly alter and modify the contained products, affecting their properties and therapeutic activity (in the case of drugs) while potentially being toxic. It is indeed true that in the field of food production, temperature plays a decisive and key role, whereas in the pharmaceutical field, products are generally not exposed to high temperatures; in fact, they are usually stored at low temperatures. For this reason, evaluating possible migration processes may not be considered as necessary in the pharmaceutical area.

In conclusion, further research in the pharmaceutical area seems advisable for future efforts.

### 4.3. Strengths and Limitations

A potential limitation of this systematic review lies in the decision to select only reviews articles. This decision was made on the basis that these reviews compile highly relevant information on the topics of interest, although it should be noted that the report was a commission report focused on the topic covered by the paper [35].

Another limitation was the large number of reviews (54 out of 80 in the case of food area, and 48 out of 52 in the case of pharmaceutical area) that met our search criteria for the selected terms of interest as TOPIC but were later found to be irrelevant based on their title or content. When using the search term “plastic*,” many of the articles found referred to plasticity instead of plastics, or to microplastics and nanoplastics. While these subjects are highly pertinent in the context of pollution, they fall outside the focus of this review.

Therefore, initially, the keyword “migration” was used only for food, and “leaching” for pharmaceuticals, in accordance with the recommendations of certain preceding articles [21]. However, the findings of this systematic review revealed no significant differences between the utilization of the two terms, as they are employed interchangeably.

## 5. Conclusions

This systematic review has focused on the migration and leaching of chemical compounds from plastic materials frequently used as containers in the food and pharmaceutical sectors. As a result, twenty-eight reviews were chosen and analyzed, addressing key aspects such as the types of migrant compounds, the analytical methodologies employed, the migration testing protocols, and the toxicological risk assessments conducted. The review revealed that phthalates, BPA, and NIAS are the most frequently studied compounds, due to their potential health risks and widespread presence in packaging materials. Chromatographic techniques, particularly those coupled with mass spectrometry, were identified as the most reliable and sensitive methods for detecting these substances, while alternative approaches such as electrochemical sensors and QuEChERS methods are gaining relevance.

It has been highlighted how important it is to control this process and to obtain robust and standardized analytical methodologies that can quantify the levels of migrants at any given time, thus ensuring that the detected concentrations remain below the regulatory limits established by institutions such as EFSA and the FDA.

This need exists in both the food and pharmaceutical sectors, although the food sector has received significantly more attention in the literature. It is indeed true that in the field of food production, temperature has been identified as the most critical factor influencing migration, whereas in the pharmaceutical field, products are generally stored at lower temperatures. However, migration can still occur during manufacturing, transport, or under accelerated stability conditions and should not be overlooked. This could explain why the migration process is more frequently investigated in the field of food science. This review underscores the importance of expanding research efforts in the pharmaceutical sector.

## Figures and Tables

**Table 1:** Summary of included studies characteristics.

Author and Year	Analytes	Food/Drugs	Toxicity Risk Assessment	Analytical Techniques	Migration Tests
Muncke, 2011 [37]	BPA	Food	YES	YES	YES
Bhogal et al., 2023 [38]	Phthalates	Food		YES	
Cao, 2010 [39]	Phthalates	Food	YES	YES	
Vinod et al., 2023 [40]	BPA	Food		YES	
Gugoasa, 2019 [41]	BPA	Food		YES	
Cao, 2012 [42]	BPA	Food		YES	
Qi et al., 2023 [43]	Phthalates	Food		YES	
Priovolos et al., 2023 [44]	BPA	Food	YES	YES	YES
Krska et al., 2012 [45]	EDCs	Food		YES	YES
Martínez-Bueno et al., 2019 [46]	NIAS	Food		YES	
Salazar-Beltrán et al., 2018 [47]	Phthalates	Food	YES	YES	
González-Sálamo et al., 2018 [48]	Phthalates	Food		YES	
Zhang et al., 2021 [49]	BPs	Food		YES	
Freitas et al., 2023 [50]	Phthalates	Food	YES	YES	
Amritha et al., 2022 [51]	Phthalates	Food	YES	YES	
Kato et al., 2021 [52]	NIAS	Food	YES	YES	YES
Urbelis et al., 2021 [53]	Leachable compounds	Food			YES
Kappenstein et al., 2012 [54]	Phthalates	Food	YES		
Maragou et al., 2024 [55]	Leachable compounds	Food	YES	YES	YES
Lopes et al., 2023 [56]	Leachable compounds	Food	YES	YES	
Pawlicka et al., 2020 [57]	NIAS	Food	YES		
Hoppe et al., 2016 [58]	Oligomers, NIAS	Food		YES	YES
Riboni et al., 2023 [3]	NIAS	Food		YES	YES
Miralles et al., 2025 [59]	NIAS	Food	YES	YES	YES
Sica et al., 2020 [36]	Leachable compounds	Food/Drugs	YES	YES	
Landrigan, et al., 2023 [35]	NIAS and others *	Food/Drugs	YES		
Kaya et al., 2022 [60]	BPs	Drug	YES	YES	
Bernard et al., 2014 [61]	Phthalates	Drug	YES	YES	

**Description:** The table displays characteristics of included studies in the systematic review including the author, year of publication, analytes included in these studies, the field are of interest (food/drug), and the different possible topics focused on them (toxicity risk assessment, analytical techniques, migration tests). Abbreviatures used: BPA: Bisphenol A, NIAS: non intentionally added substances, EDCs: endocrine disrupting chemicals. * benzotrizoles, phenols, phthalates, BPA.

## Data Availability

No new data were created or analyzed in this study.

## Appendix A. PRISMA Abstract and Review Guidelines Checklist

**Section and Topic**	**Item #**	**Checklist Item**	**Reported (Yes/No)**
**TITLE**			
Title	1	Identify the report as a systematic review.	Yes
**BACKGROUND**			
Objectives	2	Provide an explicit statement of the main objective(s) or question(s) the review addresses.	Yes
**METHODS**			
Eligibility criteria	3	Specify the inclusion and exclusion criteria for the review.	Yes
Information sources	4	Specify the information sources (e.g., databases, registers) used to identify studies and the date when each was last searched.	Yes
Risk of bias	5	Specify the methods used to assess risk of bias in the included studies.	Yes
Synthesis of results	6	Specify the methods used to present and synthesize results.	Yes
**RESULTS**			
Included studies	7	Give the total number of included studies and participants and summarize relevant characteristics of studies.	Yes
Synthesis of results	8	Present results for main outcomes, preferably indicating the number of included studies and participants for each. If meta-analysis was performed, report the summary estimate and confidence/credible interval. If comparing groups, indicate the direction of the effect (i.e., which group is favoured).	Yes
**DISCUSSION**			
Limitations of evidence	9	Provide a brief summary of the limitations of the evidence included in the review (e.g., study risk of bias, inconsistency and imprecision).	No
Interpretation	10	Provide a general interpretation of the results and important implications.	Yes
**OTHER**			
Funding	11	Specify the primary source of funding for the review.	Yes
Registration	12	Provide the register name and registration number.	Yes

**Section and Topic**	**Item #**	**Checklist Item**	**Location Where Item Is Reported**
**TITLE**			
Title	1	Identify the report as a systematic review.	p. 1
**ABSTRACT**			
Abstract	2	See the PRISMA 2020 for Abstracts checklist.	p. 1
**INTRODUCTION**			
Rationale	3	Describe the rationale for the review in the context of existing knowledge.	p. 1–3
Objectives	4	Provide an explicit statement of the objective(s) or question(s) the review addresses.	p. 3
**METHODS**			
Eligibility criteria	5	Specify the inclusion and exclusion criteria for the review and how studies were grouped for the syntheses.	2.2
Information sources	6	Specify all databases, registers, websites, organizations, reference lists and other sources searched or consulted to identify studies. Specify the date when each source was last searched or consulted.	2.1
Search strategy	7	Present the full search strategies for all databases, registers and websites, including any filters and limits used.	2.1
Selection process	8	Specify the methods used to decide whether a study met the inclusion criteria of the review, including how many reviewers screened each record and each report retrieved, whether they worked independently, and if applicable, details of automation tools used in the process.	2.3
Data collection process	9	Specify the methods used to collect data from reports, including how many reviewers collected data from each report, whether they worked independently, any processes for obtaining or confirming data from study investigators, and if applicable, details of automation tools used in the process.	2.4
Data items	10a	List and define all outcomes for which data were sought. Specify whether all results that were compatible with each outcome domain in each study were sought (e.g., for all measures, time points, analyses), and if not, the methods used to decide which results to collect.	2.4
	10b	List and define all other variables for which data were sought (e.g., participant and intervention characteristics, funding sources). Describe any assumptions made about any missing or unclear information.	N/A
Study risk of bias assessment	11	Specify the methods used to assess risk of bias in the included studies, including details of the tool(s) used, how many reviewers assessed each study and whether they worked independently, and if applicable, details of automation tools used in the process.	2.5
Effect measures	12	Specify for each outcome the effect measure(s) (e.g., risk ratio, mean difference) used in the synthesis or presentation of results.	N/A
Synthesis methods	13a	Describe the processes used to decide which studies were eligible for each synthesis (e.g., tabulating the study intervention characteristics and comparing against the planned groups for each synthesis (item #5)).	2.3
	13b	Describe any methods required to prepare the data for presentation or synthesis, such as handling of missing summary statistics, or data conversions.	2.4
	13c	Describe any methods used to tabulate or visually display results of individual studies and syntheses.	2.4
	13d	Describe any methods used to synthesize results and provide a rationale for the choice(s). If meta-analysis was performed, describe the model(s), method(s) to identify the presence and extent of statistical heterogeneity, and software package(s) used.	N/A
	13e	Describe any methods used to explore possible causes of heterogeneity among study results (e.g., subgroup analysis, meta-regression).	N/A
	13f	Describe any sensitivity analyses conducted to assess robustness of the synthesized results.	N/A
Reporting bias assessment	14	Describe any methods used to assess risk of bias due to missing results in a synthesis (arising from reporting biases).	2.6
Certainty assessment	15	Describe any methods used to assess certainty (or confidence) in the body of evidence for an outcome.	N/A
**RESULTS**			
Study selection	16a	Describe the results of the search and selection process, from the number of records identified in the search to the number of studies included in the review, ideally using a flow diagram.	3.1 + Figure 1
	16b	Cite studies that might appear to meet the inclusion criteria, but which were excluded, and explain why they were excluded.	3.1 + Figure 1
Study characteristics	17	Cite each included study and present its characteristics.	3.2
Risk of bias in studies	18	Present assessments of risk of bias for each included study.	N/A
Results of individual studies	19	For all outcomes, present, for each study: (a) summary statistics for each group (where appropriate) and (b) an effect estimate and its precision (e.g., confidence/credible interval), ideally using structured tables or plots.	N/A
Results of syntheses	20a	For each synthesis, briefly summarize the characteristics and risk of bias among contributing studies.	N/A
	20b	Present results of all statistical syntheses conducted. If meta-analysis was performed, present for each the summary estimate and its precision (e.g., confidence/credible interval) and measures of statistical heterogeneity. If comparing groups, describe the direction of the effect.	N/A
	20c	Present results of all investigations of possible causes of heterogeneity among study results.	N/A
	20d	Present results of all sensitivity analyses conducted to assess the robustness of the synthesized results.	N/A
Reporting biases	21	Present assessments of risk of bias due to missing results (arising from reporting biases) for each synthesis assessed.	N/A
Certainty of evidence	22	Present assessments of certainty (or confidence) in the body of evidence for each outcome assessed.	N/A
**DISCUSSION**			
Discussion	23a	Provide a general interpretation of the results in the context of other evidence.	4.1
	23b	Discuss any limitations of the evidence included in the review.	N/A
	23c	Discuss any limitations of the review processes used.	4.3
	23d	Discuss implications of the results for practice, policy, and future research.	4.2
**OTHER INFORMATION**			
Registration and protocol	24a	Provide registration information for the review, including register name and registration number, or state that the review was not registered.	2
	24b	Indicate where the review protocol can be accessed, or state that a protocol was not prepared.	2
	24c	Describe and explain any amendments to information provided at registration or in the protocol.	N/A
Support	25	Describe sources of financial or non-financial support for the review, and the role of the funders or sponsors in the review.	p. 1
Competing interests	26	Declare any competing interests of review authors.	p. 17
Availability of data, code and other materials	27	Report which of the following are publicly available and where they can be found: template data collection forms; data extracted from included studies; data used for all analyses; analytic code; any other materials used in the review.	N/A
		**N/A: not available.**	
